# DprA from *Neisseria meningitidis*: properties and role in natural competence for transformation

**DOI:** 10.1099/mic.0.000489

**Published:** 2017-07-12

**Authors:** Eirik Hovland, Getachew Tesfaye Beyene, Stephan A. Frye, Håvard Homberset, Seetha V. Balasingham, Marta Gómez-Muñoz, Jeremy P. Derrick, Tone Tønjum, Ole H. Ambur

**Affiliations:** ^1^​ Department of Microbiology, University of Oslo, Oslo, Norway; ^2^​ Department of Microbiology, Oslo University Hospital, Oslo, Norway; ^3^​ Faculty of Biology, Medicine and Health, Manchester Academic Health Science Centre, University of Manchester, Oxford Road, Manchester, UK; ^†^​ Present address: Lovisenberg Diaconal Hospital, Oslo, Norway.; ^‡^​ Present address: Department of Life Sciences and Health, Oslo and Akershus University College of Applied Sciences, Norway.

**Keywords:** *Neisseria meningitidis*, DprA, recombination, transformation

## Abstract

DNA processing chain A (DprA) is a DNA-binding protein that is ubiquitous in bacteria and expressed in some archaea. DprA is active in many bacterial species that are competent for transformation of DNA, but its role in *Neisseriameningitidis* (Nm) is not well characterized. An Nm mutant lacking DprA was constructed, and the phenotypes of the wild-type and Δ*dprA* mutant were compared. The salient feature of the phenotype of *dprA* null cells is the total lack of competence for genetic transformation shown by all of the donor DNA substrates tested in this study. Here, Nm wild-type and *dprA* null cells appeared to be equally resistant to genotoxic stress. The gene encoding DprA_Nm_ was cloned and overexpressed, and the biological activities of DprA_Nm_ were further investigated. DprA_Nm_ binds ssDNA more strongly than dsDNA, but lacks DNA uptake sequence-specific DNA binding. DprA_Nm_ dimerization and interaction with the C-terminal part of the single-stranded binding protein SSB_Nm_were demonstrated. *dprA* is co-expressed with *smg,* a downstream gene of unknown function, and the gene encoding topoisomerase 1, *topA*.

## Introduction

*Neisseria meningitidis* (Nm) is a human commensal and pathogen; in the absence of bactericidal antibodies it can cause meningitis and/or septicaemia [1]. Nm is competent for RecA-dependent recombination of exogenous DNA taken up by natural transformation [2, 3]. Unlike most species that are competent for natural transformation, Nm and the closely related *Neisseria gonorrhoeae* (Ng) are constitutively competent, provided that they express type 4 pili (Tfp) [4]. Members of the family *Neisseriaceae* are particular in that efficient transformation requires a 10–12 bp DNA uptake sequence (DUS) [5, 6]. By contrast, the Tfp biogenesis proteins are highly conserved and are required for DNA uptake by most bacterial species that are competent for transformation [7–10].

During transformation, incoming DNA is processed by RecA, DNA processing chain A (DprA) and single-stranded DNA-binding protein (SSB) [11–16]. DprA plays a role in the transformation in all of the bacterial species that have been examined, except *Escherichia coli* [17], but the transformability of *dprA* null mutants varies with species and DNA substrates [11–16]. In Ng, inactivation of *dprA* completely eliminated transformation of plasmid DNA, and increased RecA-dependent antigenic variation, which is the first role of *dprA* beyond transformation to be demonstrated [18]. In Nm, a *dprA* null mutant strain displayed >100-fold reduction of transformation with an unspecified substrate type, as compared to wild-type [11]. Apart from this observation, Nm DprA (DprA_Nm_) has not previously been characterized. As described in other species, DprA takes part in intracellular DNA processing, interacts with RecA, displaces SSB from ssDNA, loads RecA onto ssDNA, promotes annealing of homologous ssDNA and protects incoming DNA [19–22]. In addition, DprA selectively binds and protects ssDNA from nucleases [23]. DprA in *Streptococcus pneumoniae* (DprA_Sp_) is involved in an intracellular signalling cascade that turns off natural competence [24, 25]. In *Bacillus subtilis* DprA (DprA_Bs_) appears to increase the efficiency of RecA strand exchange during transformation and form a large multiprotein complex with RecA, SSB-B and other competence proteins [22, 26]. DprA is therefore a recombination mediator protein (RMP) [19].

Comparative genomic analysis of all known transformable bacterial species has demonstrated the ubiquitous presence of *dprA* [9]. In many species, *dprA* is part of a competence regulon [27–29]. In *E. coli*, the *dprA* gene is part of an Sxy/cAMP receptor protein regulon [30]. The genes annotated as *dprA* encode an approximately 200-residue DprA core domain, which is found in 84 % of 317 completely sequenced bacterial genomes and in some archaea [20].

3D structures have been published for DprA_Sp_, *Rhodopseudomonas palustris* DprA_Rp,_ and DprA_Hp_ [21, 31]. These DprA orthologues are all dimers, and dimerization appears to be crucial for functional activity. The core domain of several DprA homologues includes a Rossman fold, and is therefore termed the Rossman fold (RF) domain; for practical purposes, it is identical to the protein family DNA_processg_A (pfam04281) [31]. Two additional, less well-conserved domains in DprA include the N-terminal sterile alpha motif (SAM) domain and the C-terminal Zα (DLM-1) domain. In pneumococcal species, the SAM domain may regulate the activation/deactivation of competence for transformation [25]. The function of the Zα domain remains uncharacterized.

In *Haemophilus influenzae*, *dprA* is co-transcribed with the neighbouring gene *dprB* and possibly *dprC* [32]. DprB is a Holliday junction resolvase whose function overlaps with the functions of RuvC [33]. The function of *dprC* is not known. The genetic context of *dprA* genes in different bacterial species indicates a link to genes encoding topoisomerases and chromosome-segregation enzymes, but the significance of this observation has been questioned, since *dprA*_Sp_ is usually only transcribed when competence is turned on [20]. Other studies suggest that topoisomerases are also required for transformation; for example, DNA topoisomerase I may be required for transformation in *H. influenzae* and DNA gyrase (DNA topoisomerase IV) in Ng [34, 35]. Topoisomerase I is upregulated by the activation of competence in *B. subtilis* [36].

In this study, we examined the Nm wild-type and *dprA* null mutant strains with regard to competence for transformation with different DNA substrates, fitness for survival under genotoxic stress and replication efficiency. The organization and co-expression of the *dprA–smg–topA* gene cluster was investigated. DprA_Nm_ was shown to interact directly with the single-stranded binding protein SSB_Nm_. These findings shed light on the role of DprA in Nm transformation.

## Methods

### Strains and growth conditions

*Neisseria* strains were grown on GC or blood agar plates, or in CO_2_-saturated GC broth at 37 °C in 5 % CO_2_. GC plates and broth were supplemented with 1 % (v/v) IsoVitaleX. *E. coli* strains were grown in LB medium or on LB agar plates at 37 °C. When applicable, antibiotics were used at the following concentrations: 100 µg ml^−1^ ampicillin, 50 µg ml^−1^ kanamycin or 8 µg ml^−1^ erythromycin. The bacterial strains are listed in Table S1 (available in the online Supplementary Material) and the plasmids are listed in Table S2.

### Construction of Nm *dprA* and *smg* null mutant strains

To generate MC58 *dprA* null mutant strains, the *dprA* (NMB0116) locus from *Neisseria meningitidis* MC58 was amplified by PCR using the primers EH_dEX_for and EH_dEX_rev. The PCR product was cloned into the plasmid pQE-30 (Qiagen), yielding pEH2. A kanamycin resistance cassette encoding aminoglycoside 3′-phosphotransferase (*aph)* was cloned into pEH2 using a *Cla*I site in the *dprA* sequence, creating the plasmids pEH3-F with *aph* in the forward orientation and pEH3-R with *aph* in the reverse orientation. The plasmids were transformed into MC58, and the transformants were selected on GC plates with kanamycin, yielding the strains EH-MC58-001 and EH-MC58-003, respectively (Table S1). For the generation of an *smg* (NMB0117) null mutant, two PCR products from *smg* were generated with the primer pairs SF86/SF87 and SF88/SF89 and together with an *aph* cassette [37] ligated into pBluescript II SK(+), yielding pSAF51. The plasmid was transformed into MC58, the transformants were selected on GC plates with kanamycin, and the clones were confirmed by PCR and sequencing. The primers used in this study are presented in Table S3.

### Quantitative transformation

Quantitative transformation of piliated Nm cells was performed essentially as previously described [38, 39]. The DNA substrate basis was pDV4-c, a plasmid containing an *ermC* erythromycin resistance gene, the *pilG* gene, and the 12-mer DUS [39]. Linear DNA for transformation was obtained by PCR amplification of the insert of the plasmid (pDV4-c) using the primers OHA11_DUS and OHA22. The DNA concentration used in transformation was 1 ng µl^−1^. Serial dilutions were plated on plain blood agar plates and blood agar plates containing 8 µg ml^−1^ erythromycin, and the colony-forming units (c.f.u.) were counted.

### Bacterial stress testing

Nm cells from overnight plate cultures were suspended in liquid GC medium to OD_660_≈0.3 and diluted 10-fold in CO_2_-saturated GC medium containing IsoVitaleX. The cells were allowed to grow for 3 h at 37 °C with rotation. Then the cells were treated separately with 0.1 mM paraquat, 0.5 mM methanesulfonate (MMS) and 10 ng ml^−1^ mitomycin C (MMC), and further grown for 1 h with rotation at 37 °C. Tenfold serial dilutions were prepared in 1× PBS, and 50 µl aliquots of the 10^−6^ and 10^−7^ dilutions were inoculated on GC agar plates. The plates were incubated overnight at 37 °C with 5 % CO_2_ for 18 h. The colonies were counted, and the percentage survival of each strain was calculated as the ratio of the number of c.f.u. from treated cells to the number of c.f.u. from non-treated MC58 wild-type cells.

### Flow cytometry analysis

Flow cytometry analysis was performed outside the *Neisseria* biosafety level-2 (BSL-2) laboratory using the less invasive pathogen Ng, due to the serious systemic infections that can be caused by Nm, which requires a BSL-2 laboratory [40]. Colonies of Ng MS11 wild-type and *dprA* mutant strains grown for 20–24 h were resuspended in CO_2_-saturated liquid GC medium supplemented with 0.5 % (v/v) IsoVitaleX to OD_660_≈0.02. The cell suspension was diluted 10-fold with GC medium and cells grown at 37 °C overnight at 30 r.p.m. to OD_660_≈0.16. The cultures were further diluted 10 times and the cells were grown at 37 °C for four doubling times at 60 r.p.m. until OD_660_=0.14–0.18. Typically, Ng has a doubling time of 60 min at 37 °C and optimal growth conditions [41]. A 1 ml sample from the exponentially growing cultures of non-treated cells was collected and kept on ice until further processing. Rifampicin (36 µg ml^−1^) [42] and cephalexin (4 µg ml^−1^) were added to 3 ml of exponentially growing Ng cells, and the cells were allowed to grow for six additional doubling times [43]. Rifampicin inhibits the initiation of replication, but allows the current round of replication to continue to completion (replication runout), resulting in fully replicated chromosomes [44]. Cephalexin stops cell division, resulting in integer numbers of chromosomes per cell [45]. Afterwards, the treated cells and non-treated control cells were processed as described elsewhere [46]. Sample processing was also carried out using a BD LSR II flow cytometer (BD Biosciences) as described in [46], and the data obtained from the flow cytometer were analysed using FlowJo version 10 software [47].

### Bioinformatics analyses

The Nm MC58 DprA protein (DprA_Nm_) sequence was obtained from the National Center for Biotechnology Information (NCBI) and the Protein Data Bank [48]. The sequence alignments were generated using Muscle 3.7 and clustalw2 [49, 50]. blast was utilized for homology searches [51]. The taxonomy data were retrieved from the NCBI taxonomy database. The neighbourhood function of STRING was utilized to map gene organization. The Structural Classification of Proteins (SCOP) and Pfam databases were used to obtain protein domain classification data [52–54]. Phyre and I-TASSER were used to generate predicted 3D structures [55, 56]. FATCAT was used for structure alignment-based database searches [57]. The identification of single-nucleotide polymorphisms (SNPs) was conducted using mega version 6 [58]. The Virtual Institute of Microbial Stress and Survival (VIMSS) website was used to predict operons [59]. BPROM, BDGP and PPP were used for promoter prediction, while TransTermHP was used to predict terminators [60–62].

### Cloning, expression and purification of recombinant DprA_Nm_, SSB_Nm_ and SSB_Nm_Δ8C

The *dprA* gene from Nm MC58 was amplified by PCR using the primers EH041 and EH042. The gene was inserted into the expression vector pET28b(+) (Novagen) to give the plasmid pMGM1 encoding DprA with an N-terminal 6×His-tag. For overexpression, *E. coli* ER2566 carrying pMGM1 was grown in LB medium with kanamycin at 37 °C with shaking until OD_600_≈0.35 and then transferred to 18 °C with shaking. At OD_600_≈0.5, 0.25 mM of isopropyl-β-d-thiogalactopyranoside (IPTG) was added and the cells were grown overnight at 18 °C and 200 r.p.m. The cells were harvested, resuspended in a lysis buffer [50 mM NaH_2_PO_4_, 300 mM NaCl, 10 mM imidazole, 1× Complete Protease Inhibitor Cocktail (Roche), pH 8] and sonicated. The cell debris were removed by centrifugation and the cleared lysate was loaded onto a Ni-NTA column (Qiagen). The column was washed three times with a washing buffer (50 mM NaH_2_PO_4_, 300 mM NaCl, 20 mM imidazole and 0.05 % Tween; pH 8.0), and the bound protein was eluted with a sodium phosphate buffer (50 mM NaH_2_PO_4_, 300 mM NaCl, 250 mM imidazole and 0.05 % Tween; pH 8). The fraction was analysed using 10 % Bis-Tris protein gel (NuPAGE Novex Invitrogen) and 1× NuPAGE MOPS SDS running buffer (Fig. S1a). The purified protein was dialysed against a buffer containing 20 mM Tris-HCl, 300 mM NaCl and 1mM DTT (pH 7.5). The recombinant SSB_Nm_ protein was purified as previously described [63]. The C-terminally truncated SSB_Nm_ protein, SSB_Nm_Δ8C, was expressed from the *ssb*_Nm_Δ8C construct. The primers SF275 and SF276 were used to amplify the vector pSAF104 using the vector pEH1 as a template.

### Immunoblotting

Whole-cell lysates from the Nm MC58 wild-type and the Nm∆*dprA* mutant were separated by SDS-PAGE and transferred onto a polyvinylidene fluoride (PVDF) membrane. The membranes were washed with Tris-buffered saline buffer containing 0.05 % (w/v) Tween 20. Blocking was performed with non-fat dried milk. Primary antibody incubation was performed overnight at 4 °C with affinity-purified rabbit polyclonal antibodies produced against recombinant DprA_Nm_ protein. Secondary antibody incubation with anti-rabbit IgG–horseradish peroxidase conjugate was performed at 4 °C for 1 h. The immunoblots were developed using the Immun-Star WesternC Chemiluminescent kit (Bio-Rad) and visualized using a ChemiDoc XRS imager (Bio-Rad).

### Electrophoretic mobility shift assay (EMSA)

EMSA was carried out as described in [64, 65]. Briefly, 20 µl reaction mixtures containing recombinant protein and 1000 c.p.m. µl^−1^ γ-^32^P-labelled DNA substrate, in binding buffer [40 mM Tris-HCl (pH 8), 2.5 mM EDTA, 2 mM MgCl_2_, 100 mg ml^−1^ bovine serum albumin (BSA), 6 % glycerol and 1 mM DTT] were incubated for 30 min on ice. Then the samples were loaded on a 30 min pre-run 5 % native PAGE gel. In competitive EMSA, 30 nM recombinant protein was incubated with 1000 c.p.m. µl^−1^ γ-^32^P-labelled DNA for 30 min, after which cold competitor DNA was added and the mixture was incubated for an additional 30 min. Gel electrophoresis was performed using low ionic strength buffer [6.7 mM Tris-HCl (pH 8), 3.3 mM sodium acetate (pH 5.5) and 2 mM EDTA (pH 8)] at 4 °C, 100 V for 2 h with continuous buffer circulation. Autoradiography was performed for the dried gels with a PhosphorImager and image signals were quantitated using ImageQuant software (GE Healthcare).

### Co-expression analysis of *dprA*, *smg* and *topA* by RT-PCR

Reverse transcription (RT)-PCR was used to detect specific RNAs and was performed as described elsewhere [66]. Briefly, cells were grown until OD_660_≈0.6 and 5 ml culture was pelleted. Total RNA was isolated using TriZol (Invitrogen), further purified with the RNeasy kit (Qiagen), DNase-treated (Ambion) and subsequently purified by phenol/chloroform extraction and NH_4_^+^ precipitation. RNA was quantified using a NanoDrop ND-1000 (Thermo) and the integrity was inspected by agarose gel electrophoresis under native conditions. The OmniScript reverse transcription kit (Qiagen) with no RNase inhibitor was used for cDNA synthesis in a 20 µl reaction, using 2 µg RNA and 0.5 µM primer EH031. Negative controls did not contain reverse transcriptase. One microlitre of sample was added as a template to the PCR reactions for specific cDNA amplification.

### Size-exclusion chromatography assay

The direct interaction between DprA_Nm_ and SSB_Nm_ was studied by size-exclusion chromatography on a Superdex 200 10/300 GL column (GE Healthcare). Purified recombinant DprA_Nm_, SSB_Nm_ and SSB_Nm_Δ8C proteins were mixed in a buffer containing 20 mM Tris (pH 7.5), 300 mM NaCl and 1 mM DTT to a final volume of 100 µl. Before mixing, each sample was treated with 1.25 U of benzonase (Merck Millipore) to degrade any DNA present. Then the three samples, DprA, SSB, and the mixture of DprA and SSB, were independently injected into a column equilibrated with the same buffer. The proteins were eluted at a rate of 0.5 ml min^−1^ in the same buffer in 0.5 ml aliquots, and 13 µl of each fraction was separated on SDS-PAGE and stained with Coomassie blue. The concentration of proteins used in the gel-filtration assay was determined by measuring the absorbance at 280 nm and using extinction coefficients calculated from sequences using the ProtParam tool at the ExPASy website.

### Size determination of proteins

The multimeric state of SSB_Nm_ and DprA_Nm_ was studied using a Superdex 200 10/300 GL column connected to a Malvern Viscotek size-exclusion chromatograph with inline multi-angle light-scattering (SEC-MALS) system with UV, refractive index (RI) and static light-scattering (SLS) detectors for the determination of absolute molecular mass. The system was calibrated with bovine serum albumin run in the same buffer as the studied proteins, and size was estimated from the main eluting peak using Malvern software.

### Microscale thermophoresis (MST)

MST is a method for measuring molecule interaction [67]. Labelling of SSB_Nm_ and SSB_Nm_Δ8C was carried out following the manufacturer’s instructions using the Monolith NT Protein Labeling kit RED-NHS (NanoTemper Technologies), resulting in a degree of labelling (DOL) of 0.3 to 0.6. Different concentrations of DprA_Nm_ were incubated with 23.6 nM SSB_Nm_ or 25.6 nM SSB_Nm_Δ8C in 20 mM HEPES buffer (pH 7.5) containing 300 mM NaCl, 0.05 % Tween 20, 0.1 % Pluronic F-127, 0.1 % PEG 8000 and 2 mM DTT. Samples were immediately loaded into Premium coated capillaries (NanoTemper Technologies) and measured at 22 °C and 20 % MST power in a Monolith NT.115 series instrument (NanoTemper Technologies). Data analysis was performed using MO.Affinity Analysis version 2.1.3 (NanoTemper Technologies).

## Results

### Effect of deletion of *dprA o*n Nm transformability

**Fig. 1.**
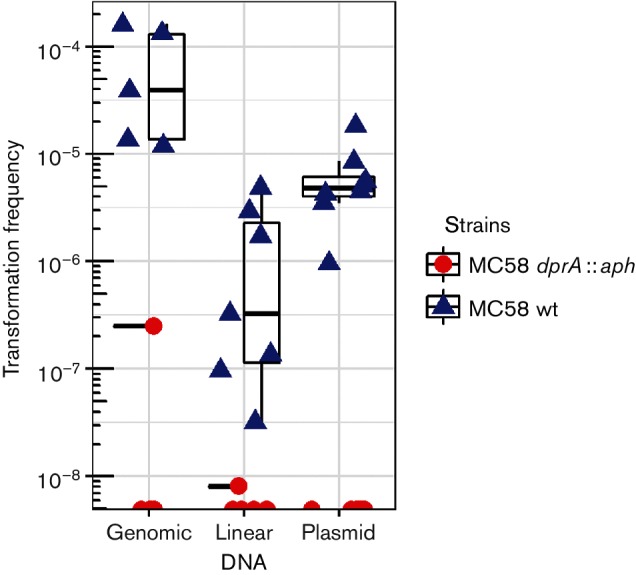
DprA is absolutely required for DNA transformation *in Neisseria meningitidis* (Nm). Variation in quantitative transformation is shown for Nm MC58 wild-type and MC58 *dprA *:: *aph* with the DUS containing genomic DNA, linear DNA and plasmid DNA substrates. The values on the *y*-axis are on a log scale. The standard deviations from at least five independent experiments are indicated by bars.

A *dprA* null mutant of Nm strain MC58 was constructed. The requirement for a functional *dprA* locus for transformation has been demonstrated in Nm [10] as well as in Ng [17]. To test the role of DprA_Nm_ in transformation with different DNA substrate conformations, wild-type and *dprA* null mutant cells were transformed with circular plasmid DNA, chromosomal DNA or PCR-amplified linear chromosomal DNA, all containing an identical *pilG *:: *kan* insert. In the wild-type background, transformation levels of 7.10×10^−5^, 1.43×10^−6^ and 6.75×10^−6^ were observed for genomic DNA, linear DNA and plasmid DNA substrate, respectively. In the *dprA* null background, the transformation rates were almost not detectable (detection limit: 1×10^−8^) (Fig. 1).

### No effect of deletion of *dprA o*n Nm DNA repair or recombination

**Fig. 2.**
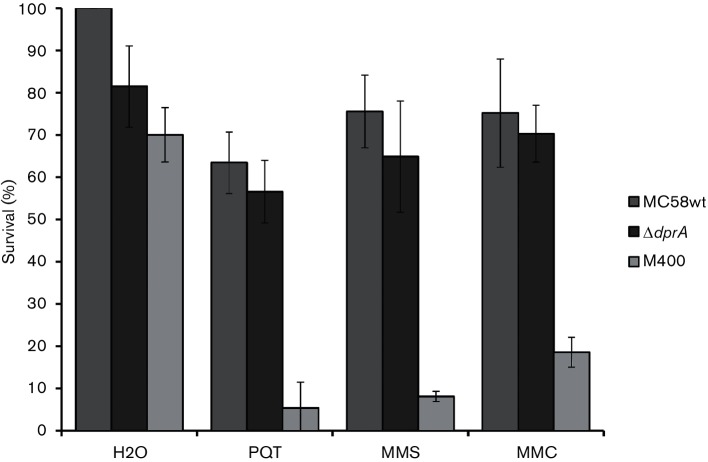
The *Neisseria meningitidis* (Nm) Δ*dprA* mutant did not show significant difference from the wild-type when exposed to DNA-damaging agents. The Nm MC58 wild-type and Δ*dprA* mutant were exposed to 0.1 mM paraquat, 0.5 m M MMS and 10 ng ml^−1^ MMC. M1080 *recA6* (i.e. IPTG-inducible *recA*, but with no IPTG added) was included as a recombination-deficient control, and showed significantly reduced survival. The standard deviations of the median from three independent experiments are indicated by bars.

Wild-type and *dprA* null cells were exposed to the DNA-alkylating agents mitomycin C (MMC) and methyl methanesulfonate (MMS), and the oxidative agent paraquat dichloride (PQT) (Fig. 2). Comparison of the survival rate between the wild-type and *dprA* null mutant cells after exposure to these DNA-damaging agents revealed no difference. However, a recombination-deficient control strain, M1080 *recA6* (M400), showed significantly reduced survival (*P*≤0.001, Student's *t*-test). This suggested that DprA_Nm_ may have little or no role in the repair of alkylating or oxidative DNA damage.

Cell-cycle progression was also assessed in Ng *dprA* null mutant strains and the wild-type strain by flow cytometry, which provided information on the DNA and protein content per cell, and the number of chromosome equivalents per cell. Both the wild-type and the ∆*dprA* mutant strains exhibited equal cell mass and contained equal numbers of chromosome equivalents, before and after rifampicin and cephalexin (CPX) treatments. However, the DNA content of the Δ*dprA* mutant cells was significantly affected after rifampicin and CPX exposure; the DNA content of the Δ*dprA* mutant cells was 242, and that of the wild-type was 277 (fluorescence in arbitrary units, AU) (*P*=0.02, Student’s *t*-test). Although it was not significant, the untreated stationary phase Δ*dprA* mutant cells also contained smaller amounts of DNA (247 AU) than the wild-type cells (276 AU) (*P*=0.109, Student’s *t*-test) (Table 1 and Figs S2 and S3).

**Table 1.** The DNA content and cell mass of individual cells derived from flow cytometer analysis

Strains	DNA per cell	Mass per cell	Relative DNA content	Relative mass
MS11wt	276	139	1.00	1.00
MS11Δ*dprA*	247	113	0.89	0.81
MS11wt^++^	277	94	1.00	1.00
MS11Δ*dprA*^++^	242	91	0.87	0.97

**^++^,** Strains treated with rifampicin and cephalexin.

### Similarity of DprA_Nm_ to well-characterized orthologues

**Fig. 3.**
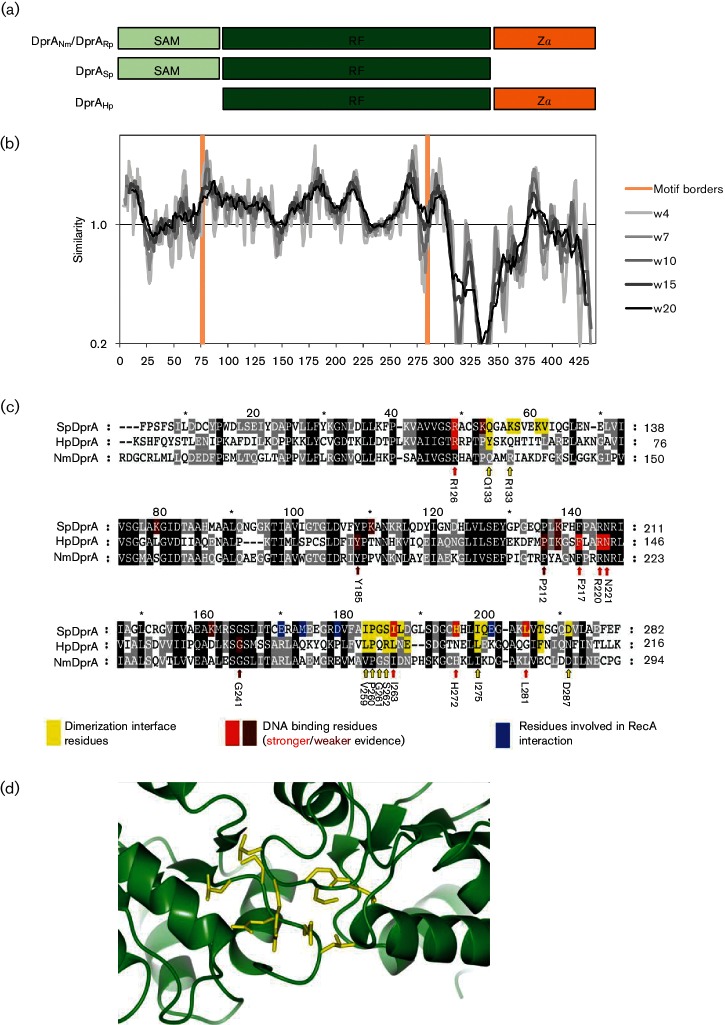
Bioinformatics comparison of DprA_Nm_ with orthologues – *Streptococcus pneumoniae* (DprA_Sp_), *Helicobacter pylori* (DprA_Hp_) and *Rhodopseudomonas palustris* (DprA_Rp_). (a) Predicted overall domain structure of all four DprA orthologues. (b) Similarity plot for DprA_Nm_ sequences with sliding window sizes (w) ranging from 4 to 20 aa. The average similarity for the whole sequence was set to 1. The borders of the three motifs are indicated with orange colour. (c) Deduced amino acid sequence alignment of the hallmark DprA domain, RF, from DprA_Sp_, DprA_Hp_ and DprA_Nm_. Experimentally suggested functional residues from DprA_Sp_ and DprA_Hp_ are coloured. Two colours mean two functions. The arrows indicate experimentally proven functional residues from other species that are conserved in DprA_Nm_. The number below each arrow shows the residue number relative to the N-terminus of DprA_Nm_. (d) Predicted monomer–monomer interaction of the DprA from Nm strain MC58. Dimerization interface residues are shown in yellow. SAM, Sterile alpha motif; Zα, a winged-helix DNA-binding motif/Z-DNA-binding domain.

The deduced amino acid sequence of DprA_Nm_ was aligned with sequences from DprA_Sp_ and *Helicobacter pylori* DprA (DprA_Hp_), revealing a high level of homology (38 and 30 % identity, respectively) at the protein level (Fig. 3). Sequence conservation among the domains was also observed (Fig. S4), albeit with some degree of variation; for example, the SAM of DprA_Nm_ had 32 % identity with the DprA_Rp_ SAM. The DprA_Nm_ RF domain had 36, 45 and 47 % sequence identity with the RFs of DprA_Hp_, DprARp and DprA_Sp_, respectively (Fig. 3a–c). The *dprA*/DprA nucleotide and protein sequences from 6 Nm strains were aligned, revealing 67 SNPs, including 47 nsSNPs (Fig. S5a). Predictions of the effect on the function of the protein using SNAP2, however, identified that 46/47 (98 %) of the SAPs were conservative. Only the SAP at position 247 was predicted to have an effect on DprA function, with a score of 45 and an expected accuracy of 71 % (Fig. S5b). Based on published structural models for DprA_Hp_ and DprA_Sp_ [21, 31], and associated biophysical data [20], we would expect the dimerization interface (termed ‘C/C’) [21] to be conserved in DprA_Nm_ (Fig. 3d). Quevillon-Cheruel *et al*. established the importance of this interface for the formation of the DNA substrate complex and transformation [21].

### DprA_Nm_ binds DNA

**Fig. 4.**
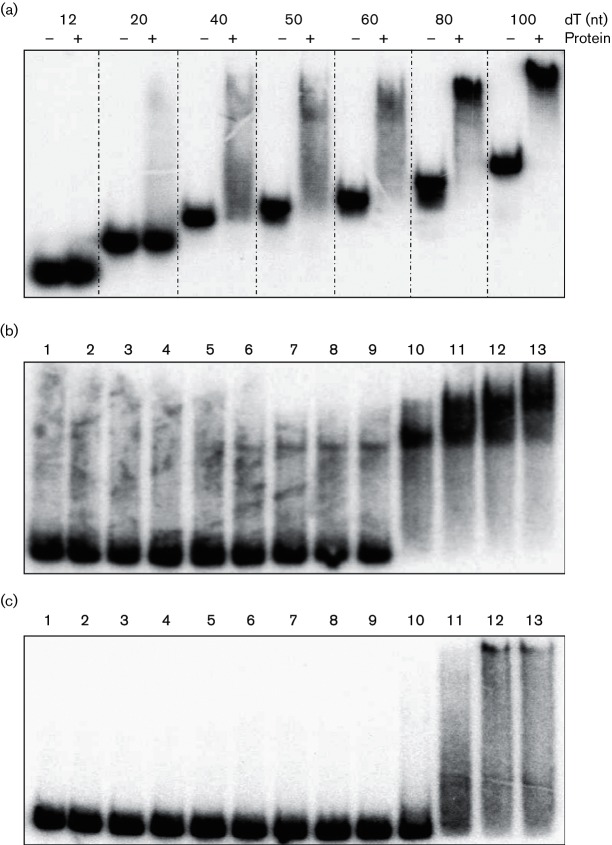
Electromobility shift assay analysis of *Neisseria meningitidis* (Nm) DprA DNA binding with DNA substrates of various lengths. Increasing binding affinity of DprA with increasing length of oligonucleotides when 30 nM of DprA was incubated with poly(T) (dT) oligonucleotide (dT12–dT100) was detected (a). DprA bound ssDNA (C80) with stronger affinity (b) compared to dsDNA (G80C80) (c), when an increasing concentration of DprA in (nM) was incubated with 1000 c.p.m. µl^−1^ of [γ^32^P]ATP-labelled ssDNA (C80) and dsDNA (G80C80), respectively. Lane 1, No protein; lane 2, 0.5; lane 3, 1.25; lane 4, 2.5; lane 5, 5; lane 6, 10; lane 7, 20; lane 8, 30; lane 9, 40; lane 10, 50; lane 11, 60; lane 12, 70; and lane 13, 80 nM protein.

EMSA was performed to analyse the affinity of recombinant DprA for single-stranded (ss) or double-stranded (ds) oligonucleotide DNA substrates. Homopolymer oligonucleotides (dT) of different lengths (dT12–dT100) were used and dT40 was sufficient for DprA to readily form a nucleoprotein complex, although the complex dissociates easily during electrophoresis (Fig. 4a). The affinity of DprA_Nm_ for the DNA substrate increased with increasing length of the ssDNA oligo, and a very stable DNA–DprA_Nm_ complex formed with dT80 (Figs 4a and S6a). Unless indicated otherwise, the EMSA experiments described below were performed with a DNA substrate 80 nt or 80 bp in length (the physical properties and sequences are presented in Tables 2 and S3). DprA_Nm_ binds ssDNA (C80) with significantly higher affinity (*P*=0.034, Student's *t*-test) (Fig. 4b) than it binds dsDNA (G80C80) (Figs 4c and S6b).

**Table 2.** The physical properties of the oligonucleotides GTB25 (DUS-containing) and C80 (without DUS), which were used as DNA substrates in the in electromobility shift assay

Physical constant	GTB25	C80
Oligonucleotide length	80	80
Molecular weight (kDa)	24.7	24.5
G+C content (%)	58	49
Melting temperature (°C)	80	76
Δ*G* (Kcal mol^−1^)*	131.1	117.2

*1 kcal=4.2 kJ.

**Fig. 5.**
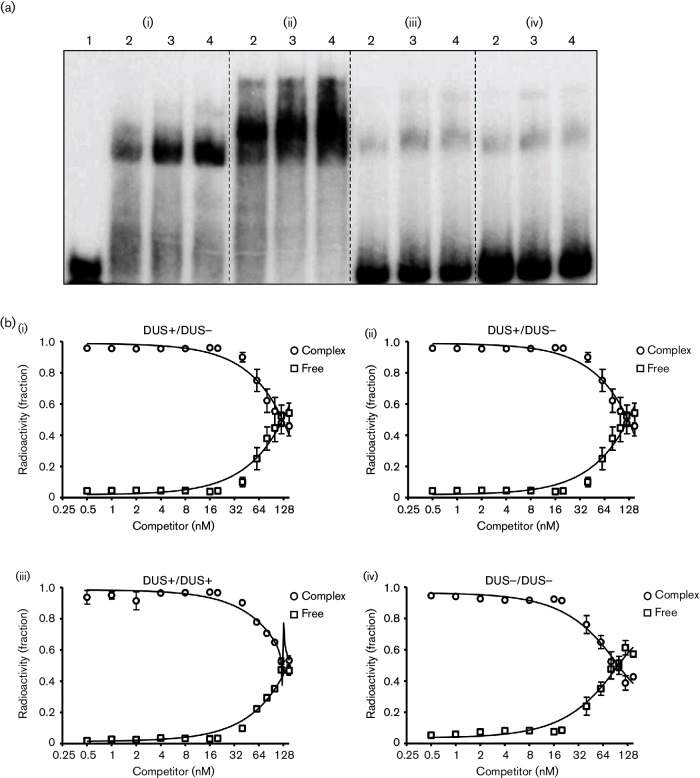
DprA exhibits sequence-independent DNA binding. (a) Representative gel images showing increasing concentration of recombinant DprA protein (nM) incubated with 1000 c.p.m. µl^−1^ of the [γ^32^P]ATP-labelled (hot) C80nt (i), GTB25 (ii), C80G80 (iii) and GTB25/26 (iv). Lane 1 – no protein. Lanes 2, 5, 8 and 11 – 20 nM. Lanes 3, 6, 9 and 12 – 30 nM. Lanes 4, 7, 10 and 13 – 40 nM. (b) Quantitation of the competitive electrophoretic mobility shift assay (for the gel images see Figs S11), where 30 nM recombinant DprA protein incubated with hot GTB25 competed with cold C80 (i), hot C80 competed with cold GTB25 (ii), hot GTB25 competed with cold GTB25 (iii), and hot C80 competed with cold C80 (iv). Note that GTB25 and GTB26 are DUS-containing oligonucleotides, whereas C80 and G80 are random-sequence oligonucleotides.

The affinity of DprA_Nm_ for C80 was compared to its affinity for an oligomer containing a DNA uptake sequence (DUS), GTB25. The results show that DprA_Nm_’s affinity for C80 and GTB25 is similar, although the mobility of the protein-bound DNA substrate during EMSA was slightly different, i.e. DprA_Nm_–C80 migrated faster than DprA_Nm_–GTB25 (Figs 5a and S7). Competitive binding assays were performed, in which pre-bound ^32^P-end-labelled GTB25 or C80 was incubated with unlabelled competitor DNA, C80 or GTB25, respectively, or vice versa. The results confirm that DprA_Nm_ binds to DUS containing GTB25 and to C80 with similar affinity [Fig. 5b(i–iv)]. Interestingly, binding to labelled oligomer was stable in the presence of up to ~15 nM competitor DNA, which is equivalent to an approximately 160-fold molar excess of unlabelled competitor. The stability of the DrpA_Nm_-bound labelled oligomer decreased when the total DNA concentration exceeded 15 nM, at which point an extra band containing labelled oligomer (B2) appeared during EMSA, with mobility in between the band (B1) and free DNA (Figs S8–S11).

### DprA_Nm_ binds SSB_Nm_ but not SSB_Nm_Δ8C *in vitro*

**Fig. 6.**
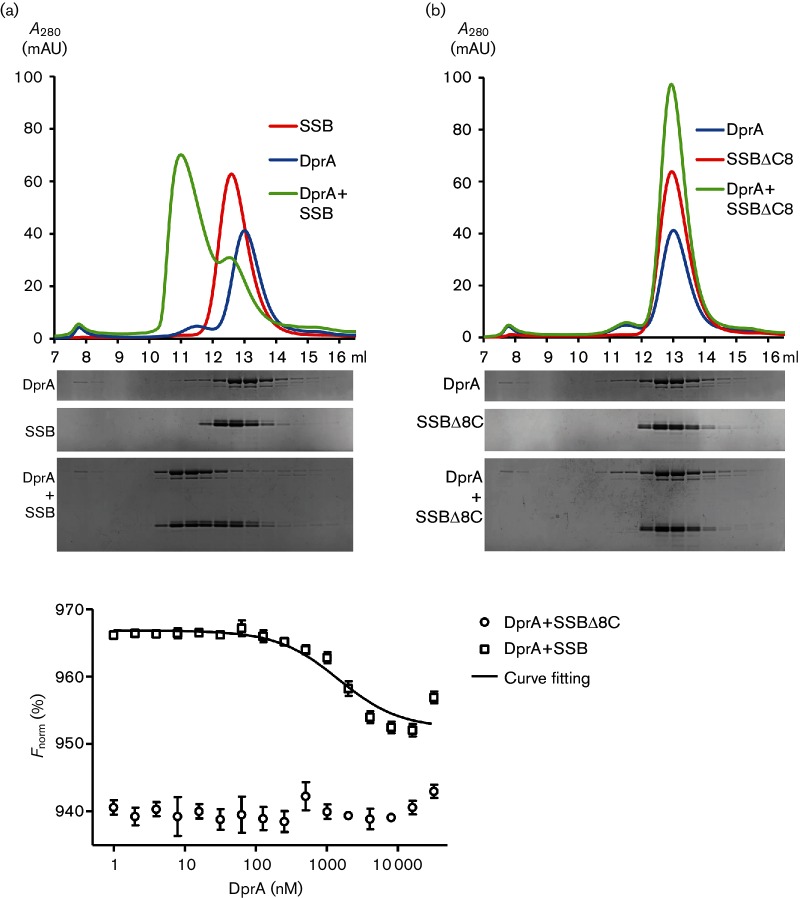
DprA_Nm_ interacts directly with SSB_Nm_, but not with SSB_Nm_Δ8C. (a) Protein–protein interaction assay by size-exclusion chromatography with 40 µM DprA_Nm_ and 80 µM SSB_Nm_. Upper panel: chromatogram *A*_280_ (mAU) versus retention volume (ml). Lower panel: SDS-PAGE of 13 µl of each 0.5 ml fraction stained with Coomassie blue, 80 µM SSB_Nm_ mixed with 40 µM DprA_Nm_ and each protein alone. The first fraction on each gel is 7 to 7.5 ml and the last fraction is 16 to 16.5 ml. (b) A similar experiment to that in (a), but with SSB_Nm_Δ8C in place of SSB_Nm._ (c) Microscale thermophoresis (MST) of DprA_Nm_ with SSB_Nm_ and with SSB_Nm_Δ8C. The ligand concentration is plotted on the *x*-axis and the normalized fit values are plotted on the *y*-axis. For the DprA_Nm_–SSB_Nm_ interaction a *K*_d_ of 1458±544 nM was calculated.

To determine whether DprA_Nm_ interacts directly with SSB_Nm_, we employed a size-exclusion chromatography column. When the mixture of DprA_Nm_ and SSB_Nm_ was injected onto the column, a new peak appeared that eluted earlier (11.0 ml) than when each of the two proteins was injected alone (Fig. 6a). Injected alone, DprA_Nm_ eluted at 13 ml and SSB_Nm_ eluted at 12.6 ml. This indicated that under the conditions used DprA_Nm_ and SSB_Nm_ are capable of forming a complex *in vitro*, without the addition of DNA (Fig. 6a). When the same experiment was performed using SSB_Nm_Δ8C, no change was seen in the elution pattern of DprA_Nm_, indicating that no detectable complex formation occurred between DprA_Nm_ and SSB_Nm_Δ8C (Fig. 6b).

The multimeric state of DprA_Nm_ and SSB_Nm_ was studied via size-exclusion chromatography with an inline SEC-MALS system under similar conditions to those during the co-size-exclusion chromatography assay. By measuring light scattering during elution of the main peaks, the software estimated a size for DprA_Nm_ of 94–96 kDa and a size for SSB_Nm_ of 91–92 kDa. This is reasonably consistent with DprA_Nm_ forming dimers with a theoretical size of 89.6 kDa and SSB_Nm_ forming tetramers with a theoretical size of 83.4 kDa. Dimerization of DprA has been reported for DprA_Sp_ [21], and tetramerization of SSB has been reported for *E. coli* [68].

In addition, using MST, the interaction between SSB_Nm_ and DprA_Nm_ was further characterized. Titration of varying concentrations of DprA_Nm_ against SSB_Nm_ gave data consistent with a single binding site and a calculated *K*_d_ value of 1458±544 nM (Fig. 6c). In contrast, the combination of recombinantly produced DprA_Nm_ and SSB_Nm_Δ8C did not result in detectable binding (Fig. 6c).

### *dprA* is co-transcribed with *smg* and *topA*

**Fig. 7.**
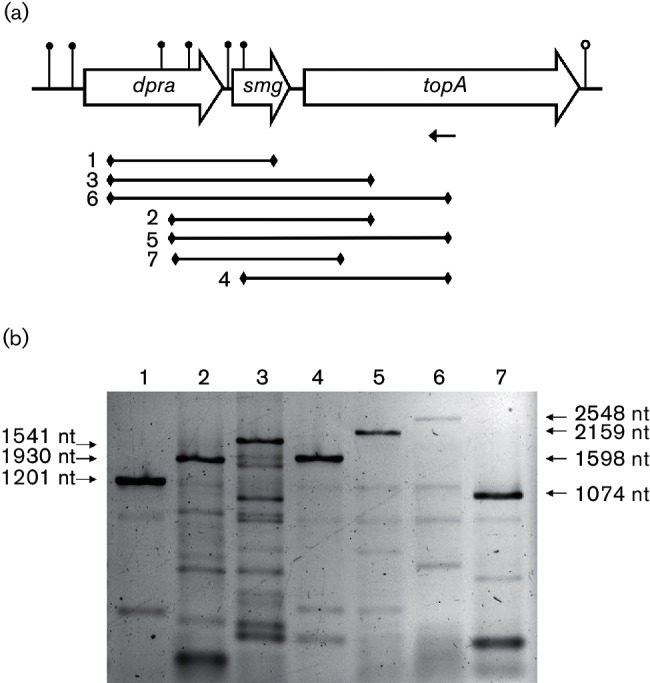
RT-PCR analysis of the *Neisseria meningitidis dprA* gene cluster. (a) Organization of the genes in the gene cluster. Open reading frames are shown as open arrows. Predicted promoters are indicated by filled circles and the predicted terminator is indicated by an open circle. The position of the primer used for reverse transcription is shown by an arrow. PCR products numbered according to the lanes in (b) are given by lines, with diamonds indicating the primer positions. The figure is not to scale. (b) Agarose gel picture of the RT-PCR products. Molecular sizes are given at the sides. The lane numbering corresponds to the numbers in (a).

Immediately downstream of *dprA*_Nm_ are the genes *smg* and *topA* (Fig. 6a). The product of the *smg* gene is a novel RNA-binding protein that acts as a translation regulator in *Drosophila melanogaster* [69]. However, the function of *smg* is unknown in bacteria, while *topA* encodes DNA topoisomerase I, a type IA topoisomerase. In the Nm MC58 genome, *dprA, smg* and *topA* were predicted by STRING to constitute an operon, with one transcription terminator on the plus strand 3′ to *topA* (Fig. 7a), one promoter on the 5′ side of *dprA* and one predicted promoter in the middle of the *dprA* ORF. Therefore, it was likely that *dprA* is co-transcribed with its neighbouring genes. Consistent with this observation, non-quantitative RT-PCR generated PCR products spanning *dprA*, *smg* and *topA* (Fig. 7b), while negative control reactions exhibited no detectable PCR product of similar size (data not shown). The *dprA* and *topA* genes are also co-localized in representative genomes from 14 of 24 bacterial phyla (Table S4). In *Myxococcus xanthus*, the *dprA* and *topA* genes are fused. *dprA–smg* co-localization is also found in other *Neisseria* species and in some *Betaproteobacteria* and *Gammaproteobacteria*. However, *smg* orthologues are only found in *Betaproteobacteria* and *Gammaproteobacteria*, with representation in *E. coli*, *B. subtilis* and *Vibrio cholerae*. In *S. pneumoniae*, *dprA*, *topA* and *smg* do not map to the same chromosomal region [70].

## Discussion

Tang and colleagues previously reported that disruption of the *dprA* locus substantially reduces Nm transformability [11]. Beyond that observation, this study is the first primary characterization of Nm DprA interaction with SSB to our knowledge. With the important exception recently reported in Ng that DprA appears to enhance pilin antigenic variation [18], DprA is dedicated to transformation, and it has been suggested that the presence of a *dprA* gene is a distinctive feature of naturally transformable species [9]. DprA is required for transformation in Nm [11] and Ng [18]. Natural transformation is a widely distributed mechanism for the acquisition of DNA and genetic recombination in many bacterial genera [9]. The competence machinery actively processes exogenous dsDNA and takes up the internalized ssDNA to replace homologous (or partially homologous) chromosomal sequences in a mechanism catalysed by RecA, with the help of accessory factors such as DprA [13, 36]. DprA is ubiquitous in the microbial domain.

In this study, transformation of Nm *dprA* null mutant cells with plasmid, linear and chromosomal DNA was tested. No transformation of *dprA* null mutant cells with any donor DNA was detected. When *dprA* null mutants were exposed to agents inducing DNA double-strand breaks, alkylation and oxidation, no difference in survival was observed relative to the wild-type (Fig. 2), which is consistent with studies of DprA mutants in other species [17]. Using flow cytometry, the overall DNA content of the Ng Δ*dprA* mutant was significantly lower than that of the MS11 wild-type after antibiotic treatments, which might suggest impaired DNA replication in *dprA* mutant cells. However, the *dprA* null mutant showed no difference in cell mass (Table 1) or chromosome equivalents per cell compared to the wild-type (Figs S2 and S3). Therefore, our findings show that *dprA* is necessary for transformation, irrespective of DNA substrate conformation, but is not required for DNA repair or cell viability.

The predicted 3D structure of the DprA_Nm_ N-terminal and C-terminal domains showed clear similarities to that of the SAM and Zα domains, respectively (Fig. 3a). In particular, the sequence similarities among the SAM and RF domains are higher (Fig. 3b). The functions of the accessory domains, SAM and Zα, in DprA_Nm_ are not yet known. Several amino acids in the dimerization interface of DprA_Hp_ and DprA_Sp_ were conserved in DprA_Nm_ (Fig. 3c, d). We therefore propose that DprA_Nm_ has a dimerization interface localized in the same region as DprA_Hp_ (Fig. 3d) [21]. Dimerization is a feature of all of the other DprA proteins described to date [20–22, 31]. Furthermore, a high level of homology is observed in the suggested DNA-binding motifs of DprA_Nm_, DprA_Sp_ and DprA_Hp_ [21, 31] (Fig. 2c). In DprA_Sp_, the SAM domain plays a role in intracellular signalling and regulation of competence [25]. Consistent with the constitutive competence of Nm, the SAM domain in DprA_Nm_ lacks amino acid residues that confer the induction of competence in Gram-positive bacterial species (Fig. S4). Generally, SAM domains are functionally diverse, playing roles in protein–protein interactions, DNA or RNA binding, or post-translational protein modification [71].

DprA orthologues are generally involved in DNA processing and only bind ssDNA, or ssDNA and dsDNA [20–23, 72]. The current model of transformation in Gram-positive bacteria implies the entry of ssDNA into the cytoplasm [73, 74]. For Gram-negative bacteria, and specifically *Neisseria* spp., there is more ambiguity in the mode of DNA entry [75], and both ssDNA and dsDNA have been reported to enter the cytoplasm [76]. The Zα domain of DprA_Hp_ binds dsDNA [72]. DprA_Sp_ only binds ssDNA [20], and does not contain the Zα domain. Here, DprA_Nm_ formed a very stable protein–DNA complex with dT80, while the minimal required oligonucleotide length is dT40, which is similar to what was reported for DprA_Sp_ [20]. On the other hand, the protein–DNA complex formed by DprA_Nm_ had greater mobility during EMSA than DNA bound by DprA_Sp_ [21]. Comparing the affinity for the DNA substrates C80 and G80C80, DprA_Nm_ exhibited stronger affinity for the ssDNA than for the dsDNA (Fig. 4b, c), which is similar to DprA_Hp_ [72].

In *Neisseria*, the DUS mediates enhanced DNA uptake in transformation [77]. Although DprA_Nm_ does not selectively bind DUS, DprA-GTB25 and DprA-C80 complexes may be structurally distinct, given their distinct mobility during EMSA [Fig. 5a(i, ii)]. C80 and GTB25 are different in their physical constants (Table 2), providing a possible explanation for distinct electrophoretic mobility [78]. Further investigation by competitive EMSA confirmed the DUS-independent DNA binding of DprA; that is, the DUS containing GTB25 did not out compete C80 in complex with DprA more than C80 did GTB25 in complex with DprA. Interestingly, the complex formed at 30 nM DprA and 16 nM DNA [Fig. 5b(i–iv)] was extremely stable. We interpret these data to indicate that optimal binding is observed with dT80 (not dT40) and a 2 : 1 molar ratio of protein to DNA. This is consistent with the conclusion that DprA binds DNA as a dimer and is supported by the fact that DprA_Sp_ with mutations in the dimerization interface fails to bind DNA *in vitro* and fails to support efficient transformation *in vivo* [21].

Nm *dprA* null mutant cells did not display altered growth, replicative potential or survival under stress, but exhibited a total inability to be transformed, irrespective of DNA conformation. DprA is essential for transformation in Nm, Ng and *S. pneumoniae* [18, 23]. However, in *B. subtilis*, *dprA* is not stringently required for DNA transformation, as there is redundancy between the RecF and DprA pathways [79]. The classical RMPs in *E. coli* are the RecF pathway proteins [Rec(F)OR] [22]. The RecBCD holoenzyme plays a similar role [80]. The interaction and expression of RMPs in neisserial transformation have previously been discussed, before the role of DprA in transformation was known. Mutations in the Ng RecF pathway of recombination do not affect transformation [81]. This could mean that in *Neisseria*, DprA is the RMP of transformation, while RecF is the RMP of the other recombination. Given such a model, the division of labour between DprA and RecBCD, which is required for transformation in Ng [81], is still an open question.

*In vivo*, DprA_Bs_ co-localizes with SsbB [26], and *in vitro* the C-terminal residue M238 was shown to mediate DprA_Sp_ interaction with RecA [82]. Here, a direct interaction between DprA_Nm_ and SSB_Nm_ was demonstrated (Fig. 6a, c). The interaction was abolished when the last eight C-terminal residues of SSB_Nm_ were deleted. This suggests that the site for interaction with DprA_Nm_ is located in the C-terminal part of SSB_Nm_ (Fig. 6b, c). However, the exact SSB_Nm_ residues mediating the interaction with DprA_Nm_ have not yet been defined. The DprA_Nm_ site for interaction with SSB_Nm_ also remains an obvious question to investigate.

In Nm, the *dprA* gene is located directly upstream of *smg* and *topA* (Fig. 7a), and co-transcription of these three genes was detected (Fig. 7b). Nm only contains one DNA topoisomerase I, the *topA* locus, which is essential, as expected. *Hae. influenzae* contains two genes encoding DNA topoisomerase I, and one of these genes, *topA*, is required for genetic competence [34]. Operons are most often co-regulated genes with related functions that are transcribed into a polycistronic mRNA [83]. Some operons encode genes that are not part of the same functional pathway but are usually functionally related. Based on the finding that the *topA* gene product was detected by immunoblotting in a *dprA* null mutant strain, it is likely that *topA* is also transcribed independently from *dprA* (Fig. S1b). DNA topoisomerase I is known to be required for competence for DNA transformation in *H. influenzae.* However, *topA* and *smg* orthologues are not recognized partners of competence regulons, as *dprA* is [27, 28, 84, 85], and topoisomerases are recognized to be required for the unwinding of DNA in replication and transcription. The function of *smg* remains elusive, and is an interesting subject for further study. Further studies on the possible roles of these components in transformation and in other processes are therefore warranted.

DprA_Nm_ has been poorly described. Here, we have shown that it is similar to DprA orthologues and have demonstrated an absolute requirement for Nm *dprA* in transformation irrespective of DNA substrate conformation. DprA_Nm_ consists of three domains that are also present in other DprA orthologues, and functional residues are conserved. We identified a *dprA–smg–topA* operon in Nm, and this gene organization is widely conserved in bacteria. Our data demonstrate that DprA_Nm_ preferentially binds ssDNA, with lower affinity for similar-size dsDNA, but has no specificity for DUS-containing DNA. Dimerization of DprA is essential in order to form a stable protein–DNA complex. We have also demonstrated direct interaction between DprA_Nm_ and SSB_Nm_ linked to the C-terminal part of SSB_Nm_. The ubiquity of the *dprA–smg–topA* gene cluster leads to the question of whether co-transcription of these genes is not only present in Nm. The functional significance of this operon and gene cluster also remains an open question. Further exploration of the functions and interplay of the components of recombination and their interaction with DNA in the *Neisseria* and other species should also still be an interesting area for researchers in the future.

## Supplementary Data

1673 Supplementary File 1
