# Supplementary material for: DprA from Neisseria meningitidis: properties and role in natural competence for transformation
Source: Microbiology (Reading). 2017 Jul 12;163(7):1016–29. doi: 10.1099/mic.0.000489 (PMC5817196; doi:10.1099/mic.0.000489)
Supplement: Supplementary File 1 [file mic-163-1016-s001.pdf]

Supplementary material

DprA from *Neisseria meningitidis*: properties and role in natural competence

Eirik Hovland[1Θ], Getachew Tesfaye Beyene[1], Stephan A. Frye[2], Håvard Homberset[1], Seetha V. Balasingham[2], Marta Gómez-Muñoz[1], Jeremy P. Derrick[3], Tone Tønjum[1,2*] and Ole H. Ambur[2§]

[1]Department of Microbiology, University of Oslo; [2]Department of Microbiology, Oslo University Hospital, Oslo, Norway; [3]Faculty of Biology, Medicine and Health, Manchester Academic Health Science Centre, The University of Manchester, Oxford Road, Manchester U.K.

*Corresponding author

[Θ] Current address: Lovisenberg Diaconal Hospital, Oslo, Norway

[§]Current address: Department of Life Sciences and Health, Oslo and Akershus University College of Applied Sciences, Norway

Table S1. Bacterial strains used in this study

Strain	Relevant genotype	Source
E. coli		
ER2566	*fhuA2 lacZ::T7 gene1 [lon] ompT gal sulA11 R(mcr-73::miniTn10*--Tet^{S})*2 [dcm] R(zgb-210::Tn10*--Tet^{S}) *endA1 Δ(mcrC-mrr)114::IS10*	NEB
N. meningitidis		
MC58	Serogroup B, isolated in the UK 1983	[1]
EH-MC58-001	MC58 *dprA::aph*$_{\text{forward}}$	This study
EH-MC58-003	8013 *dprA::aph*$_{\text{reverse}}$	This study
M400	MS11 subclone, *recA*6 (*tetM*)	[2]
MC58Δ*smg*	MC58 *smg::aph*	This study

Table S2. Plasmids used in this study

Plasmid	Relevant genotype	Source
pQE-30	Expression vector based on a T5-promoter-driven system, His tag, Amp^{r}	Qiagen
pET-28b(+)	Kan^{r}, with an N-terminal His•Tag®/thrombin/T7•Tag® configuration plus an optional C-terminal His•Tag sequence	Novagen
pBlueScript II SK+	General cloning vector, Amp^{r}, abbreviated pBS+	Stratagene
pMGM1	pET-28b(+) containing *dprA*$_{\text{MC58}}$ with N-terminal cleavable His-tag	This study
pEH2	pQE-30 containing *dprA*$_{\text{MC58}}$	This study
pEH3-F	pEH2 *dprA*$_{\text{MC58}}$*::aph*$_{\text{forward}}$	This study
pEH3-R	pEH2 *dprA*$_{\text{MC58}}$*::aph*$_{\text{reverse}}$	This study
pSAF51	pBSK+ harbouring *smg*$_{\text{MC58}}$*::aph*	This study
pDV4-c	pBSK+ harbouring *pilG::*mTnErm w/ three *Nla*IV sites removed and DUS 5′ to the insert	[3]

Table S3. Primers and oligonucleotides used in this study.

Oligo name	Sequence 5'→3'
Primers for generation of pEH2	
EH_dEX_for	GCGGATCCATGACAGAGGACGAACGT
EH_dEX_rev	GCAAGCTTTCAAGTTCGGATACGCTGGTAT
Primers for expression of DprA recombinant protein	
EH041_Forward	TAcatatgACAGAGGACGAACGTTTCG
EH042_Reverse	ATCTCGAGTCAAGTTCGGATACGCTGGT
Primers for construction of* smg *null mutant strain	
SF86	TCTTGGACAGGATGGGTTTC
SF87	GGCAGGTATCGAAATCTTGG
SF88	ATTGCTCAACAGCTCCGAAT
SF89	ATAAAAGCAGCAGGGTCAGC
Primers for construction of* $SSB_{Nm}\Delta 8C$ *strain	
SF275	CCGCCGCCCCGGTCGAGTGAGAGCTCAATTAGCTGAGCTTGGACT
SF276	AAGCTCAGCTAATTGAGCTCTCACTCGACCGGGGCGGCGGCA
Reverse transcription primer	
EH031	TTTTGCTGTCTTCGCCTTCT
Reverse transcription PCR primers	
EH003	GACGACATCCTGAACGAATGCCC
EH006	GCGGCTGATGAGTTGGTATT
EH007	CACAGTAAAGGCTGCCACAA
EH033	CGCTCTTGTTTGCCCATAAA
EH036	AAGATTTCGGCAAGTCGTTG
EH038	GTCCAATATTCCTGCGCTTC
OHA2180	ATATCGACGGTGGTTTGGTC
Primers for generation of DNA constructs for transformation	
OHA11_DUS	ACGACTCGAGATGCCGTCTGAAATGGCTAAAAACGGAGGAT
OHA22	TAGAGAATTCTCAGGCGACCACGTTGCC
Sequencing primers	
PR2488	GTAAAACGACGGCCAGT
PR2487	AGCGGATAACAATTTCACACAGGA
Oligonucleotides used in electromobility shift assays	
GTB25*	GCCGTCTGAAagctctagGCCGTCTGAAacgtcagGCCGTCTGAAagctctagG CCGTCTGAAacgtcagGCCGTCTGAA
GTB26*	TTCAGACGGCctgacgtTTCAGACGGCctagagctTTCAGACGGCctgacgtTTC AGACGGCctagagctTTCAGACGGC
C80	GCTGATCAACCCTACATGTGTAGGTAACCCTAACCCTAACCCTAAG GACAACCCTAGTGAAGCTTGTAACCCTAGGAGCT
G80	AGCTCCTAGGGTTACAAGCTTCACTAGGGTTGTCCTTAGGGTTAGG GTTAGGGTTACCTACACATGTAGGGTTGATCAGC

***DUS in Block letters and the intervening 8 nucleotides in small letters.

Table S4. Number of species within each bacterial phylum, in which *dprA* and *smg* orthologs are present (number of species with BLAST hits of a homology search using the $DprA_{Nm}$ deduced amino acid sequence). Bacterial phyla in which *dprA*, *smg* and *topA*, are colocalized on the genome (as reported by STRING).

**Bacteria**	***dprA* present**	***smg* present**	***dprA-topA* genomic neighbourhood**	***dprA-smg* genomic neighbourhood**
Proteobacteria	2186	754	X	X
Firmicutes	1491		X	
Spirochaetes	72		X	
Chlamydiae / Verrucomicrobia group	17		X	
Synergistetes	13		X	
Chloroflexi	19		X	
Thermotogae	15		X	
Acidobacteria / Fibrobacteres group	10		X	
Fusobacteria	33		X	
Dictyoglomi	2		X	
Elusimicrobia	2		X	
Actinobacteria	555			
Cyanobacteria	72			
Bacteroidetes / Chlorobi group	248			
Tenericutes	24			
Aquificae	11			
Deinococcus-Thermus	19			
Thermodesulfobacteria	2		X	
Nitrospirae	7			
Planctomycetes	10		X	
Armatimonadetes				
Caldiserica	1			
Chrysiogenetes	1			
Deferribacteres	3			
Gemmatimonadetes	1		X	

(a)

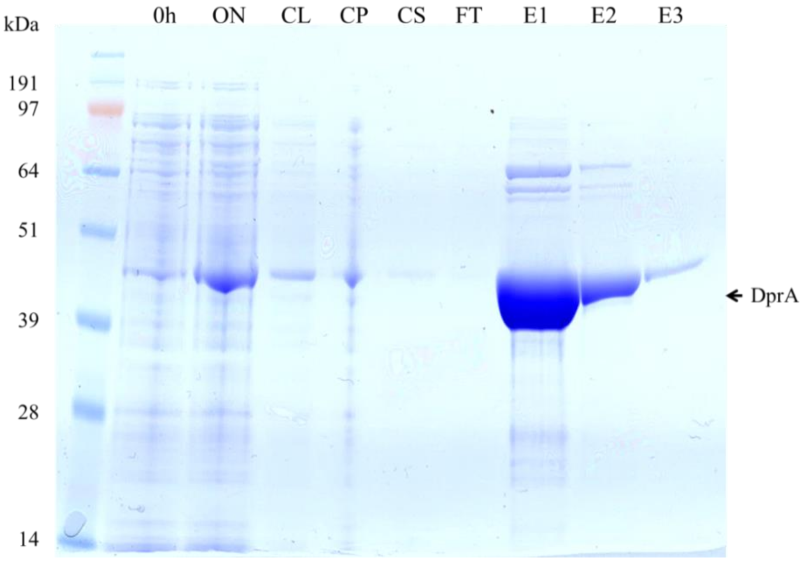

(b)

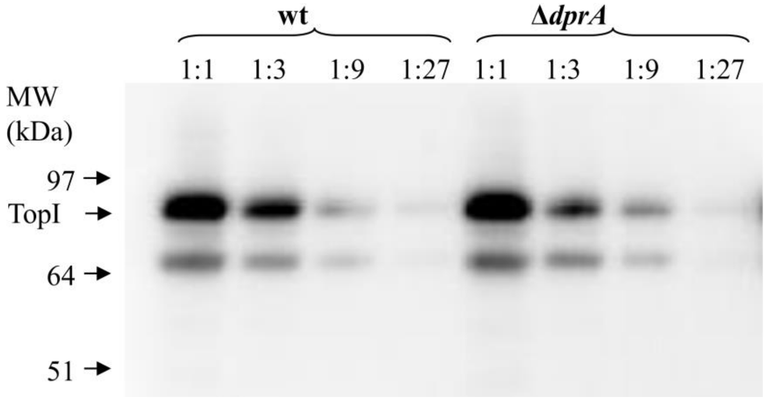

Figure S1. Protein purification of $DprA_{Nm}$ with an N-terminal 6xHis-tag and Nm MC58 whole cell lysates immunoblot. Coomassie blue stained NuPAGE^{TM} Novex^{TM} 10% Bis-Tris protein gel image of culture before induction (0h), ON culture after IPTG induction (ON), cell lysate (CL), cell pellet (CP), cell supernatant (CS), flow through Ni-NTA slurry column (FT), first (E1), second (E2) and third (E3) elution **(a)**. Immunoblot of Nm whole cell lysates using antibody against TopI, four dilution levels of each lysate were used (1:1-1:27) **(b)**.

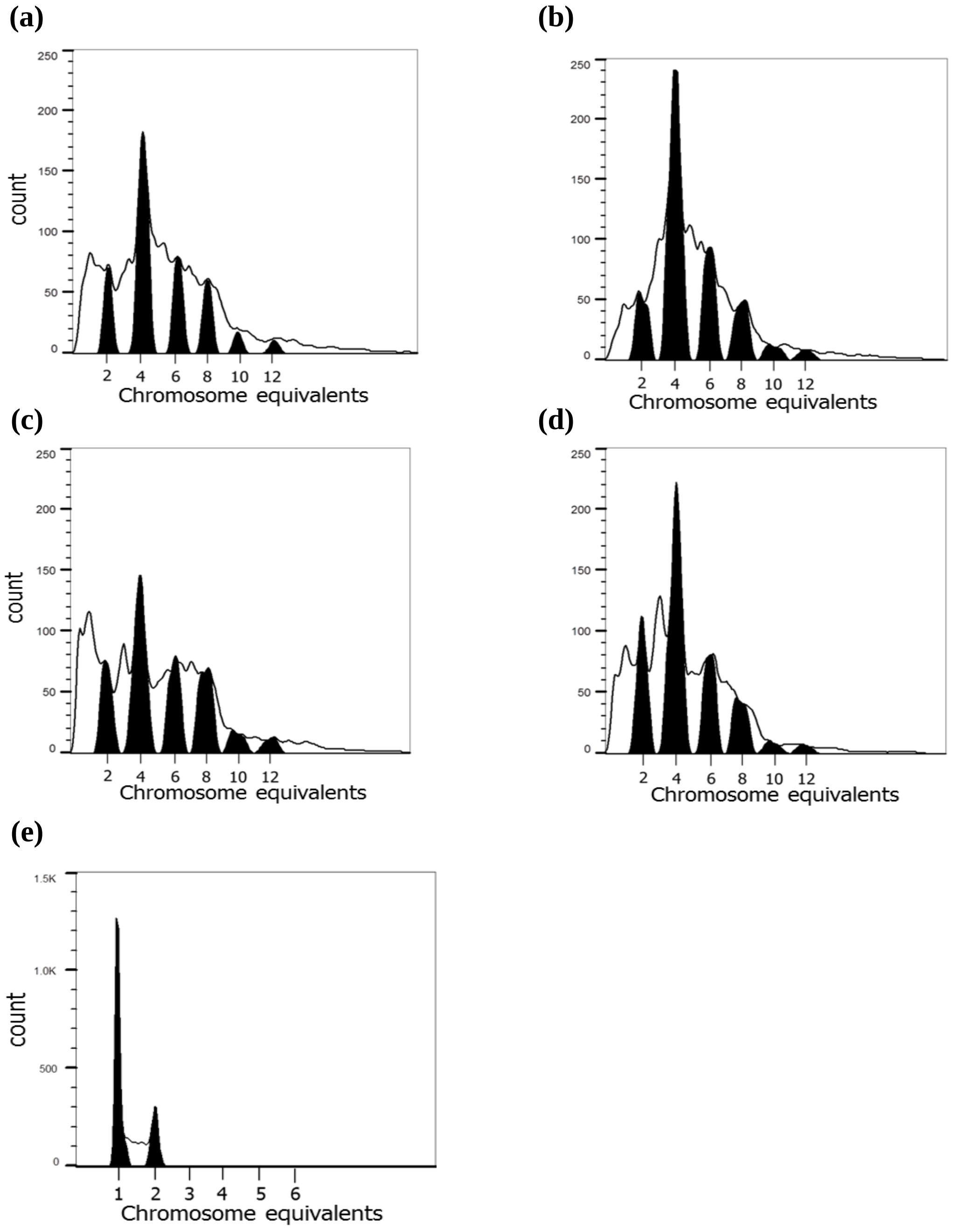

Figure S2. DNA measurement by flow cytometry. Histograms of the DNA content measured by fluorescence intensity of Hoechst 33258 stained wildtype Ng **(a & c)**, Δ*dprA* Ng **(b and d),** and the control strain slowly growing *E. coli* CM735 **(e)**. Chromosome equivalents were determined from the untreated stationary phase **(a & b),** and Rif and CPX treated cells **(c & d)**. The x-axis shows fluorescence levels indicating the amount of DNA content per cell count, calibrated to *E. coli* strain CM735 as standard. The Y-axis represents the cell count.

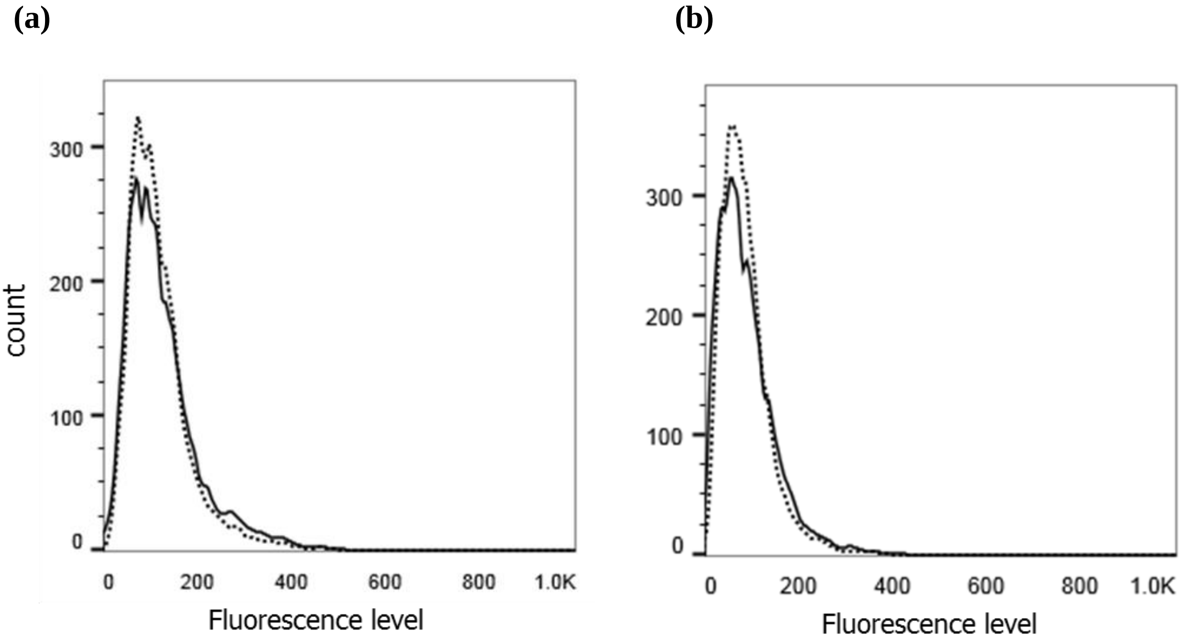

Figure S3. Protein measurement by flow cytometry. Histograms of total protein measured by fluorescence of FITC labeled proteins. The X-axis gives the fluorescence intensity and the Y-axis designates the event counts. Ng wildtype cells (continuous line) and delta-dprA cells (dotted line) from exponential culture untreated (a) and treated with 40 µg/ml Rif and 4 µg/ml CPX (b) are shown.

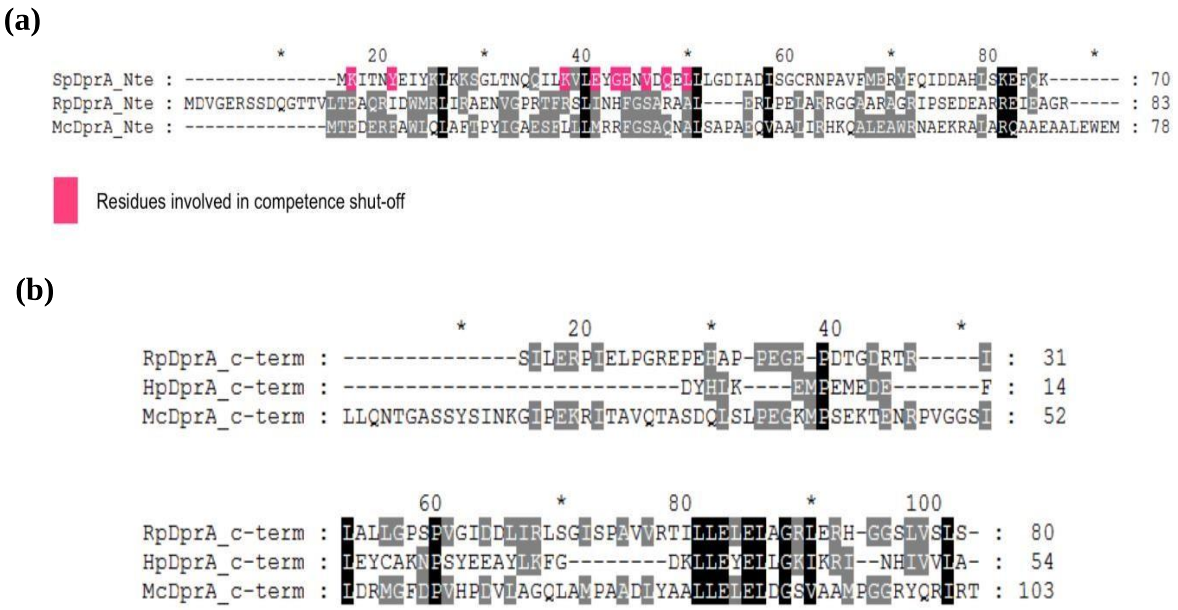

Figure S4. Amino acid comparison of Nm DprA with orthologs; *Streptococcus pneumoniae* (DprA), *Helicobacter pylori* (DprA), and *Rhodopseudomonas palustris* (DprA). (a) Deduced amino acid sequence alignment of the N-terminal domains of $_{Sp}$DprA, $_{Rp}$DprA, and $_{Mc}$DprA. (b) Deduced amino acid sequence alignment of the C-terminal domains of $_{Mc}$DprA, $_{Hp}$DprA, and $_{Rp}$DprA.

(a)

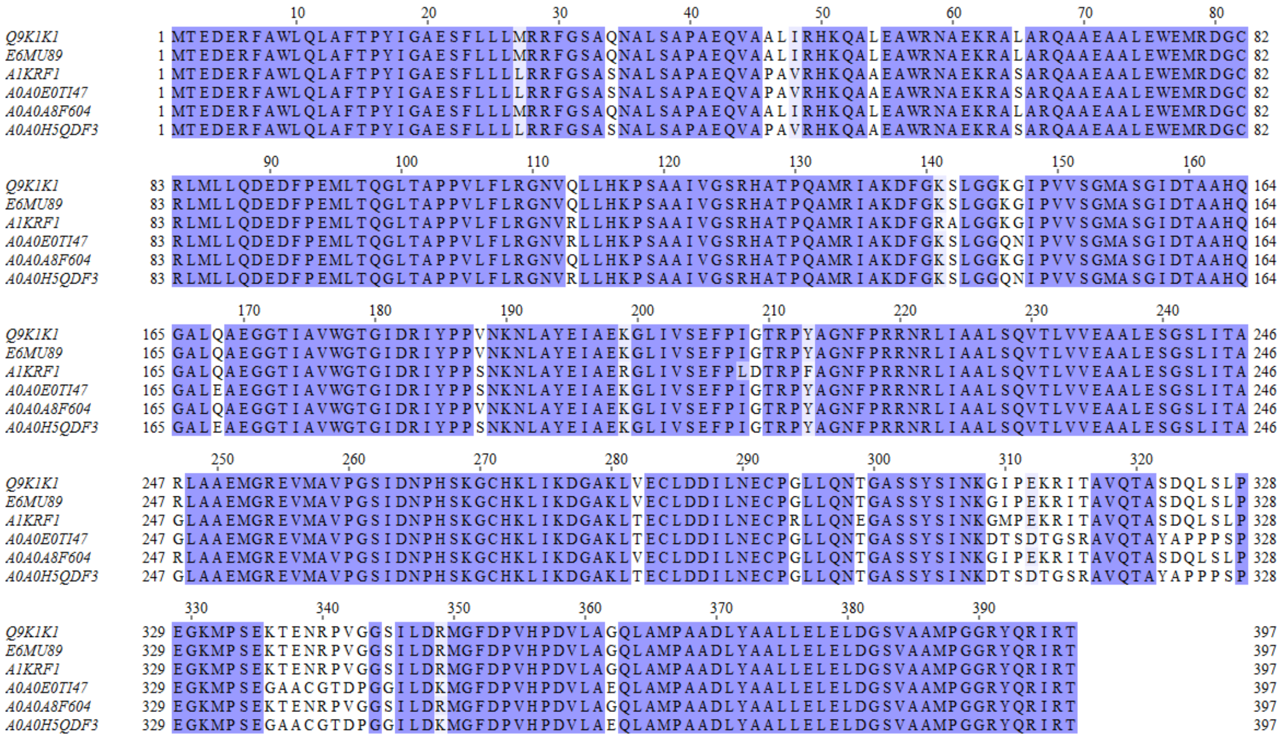

(b)

Strains	27	34	46	47	48	54	65	113	141	142	146	147	168	188	199	208	209	213	247	282	294	299	309	310	311	312	313	314	315	316	322	323	324	325	326	327	336	337	338	339	340	341	342	343	345	349	362
N. meningitidis MC58	M	Q	A	L	I	L	L	Q	K	S	K	G	Q	V	K	I	G	Y	R	V	G	T	G	I	P	E	K	R	I	T	S	D	Q	L	S	L	K	T	E	N	R	P	V	G	S	R	G
N. meningitidis H44/76	.	.	.	.	.	.	.	.	.	.	.	.	.	.	.	.	.	.	.	.	.	.	.	.	.	.	.	.	.	.	.	.	.	.	.	.	.	.	.	.	.	.	.	.	.	.	.
N. Meningitidis FAM18	L	S	P	A	V	A	S	R	R	A	.	.	.	S	R	L	D	F	G	T	R	E	.	M	.	.	.	.	.	.	.	.	.	.	.	.	.	.	.	.	.	.	.	.	.	.	.
N. Meningitidis M04-240196	L	S	P	A	V	A	S	R	.	.	Q	N	E	S	.	.	.	.	G	T	.	.	D	T	S	D	T	G	S	R	Y	A	P	P	P	S	G	A	A	C	G	T	D	P	G	K	E
N. meningitidis LNP21362	.	.	.	.	.	.	.	.	.	.	.	.	.	.	.	.	.	.	.	.	.	.	.	.	.	.	.	.	.	.	.	.	.	.	.	.	.	.	.	.	.	.	.	.	.	.	.
A0A0H5QDF3 *N. Meningitidis*	L	S	P	A	V	A	S	R	.	.	Q	N	E	S	.	.	.	.	G	T	.	.	D	T	S	D	T	G	S	R	Y	A	P	P	P	S	G	A	A	C	G	T	D	P	G	K	E

Figure S5. Multiple sequence alignment of the DprA proteins (identified by their accession number on the left) from six Nm strains were aligned using Jalview open source multiple sequence alignment program, the residues were shaded based on percentage identity **(a)**. The SAPs positions identified in the six strains of Nm DprA are shown in the top row; only the SAPs at positions 247 is predicted to have intermediate effect on the DprA function **(b)**.

(a)

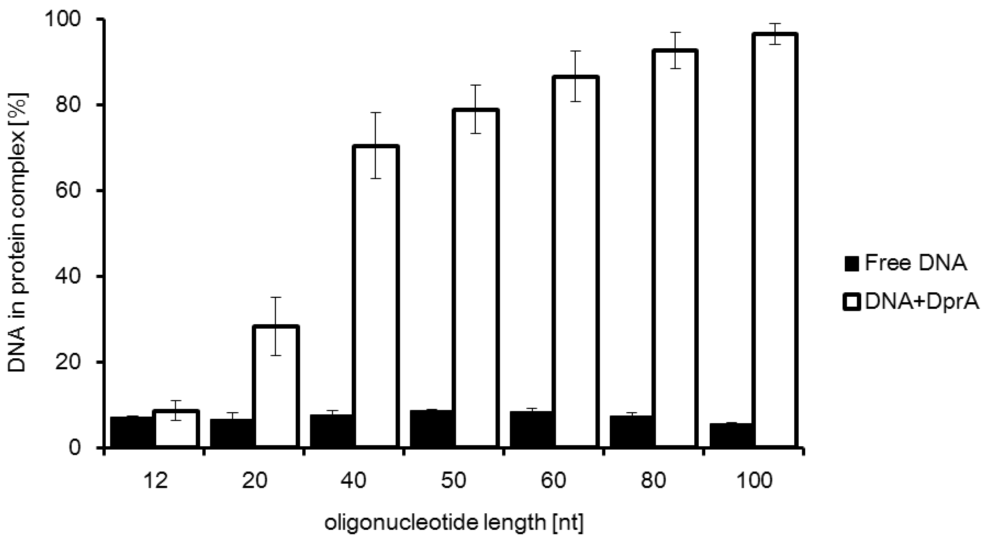

(b)

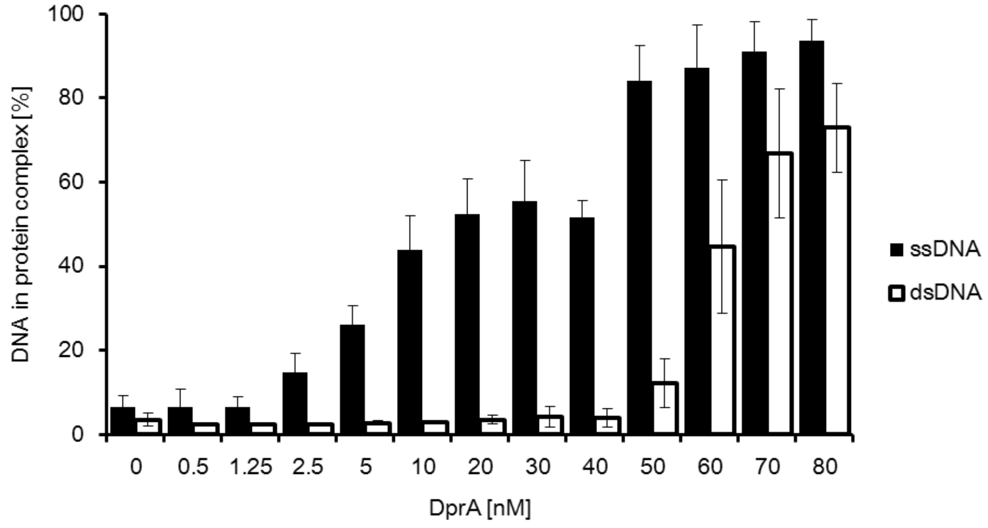

Figure S6. Quantitation of DprA DNA binding activity. (a)30nM of DprA incubated with the indicated length of 1000 CPM/µl of [γ^{32}P]ATP labeled dT12 - dT100. (b) increasing concentration of recombinant DprA protein in (nM) incubated with 1000 CPM/µl of [γ^{32}P]ATP labeled ssDNA (C80) and dsDNA (G80C80). DprA binding activity is expressed as a fraction of DNA–DprA complexes at the indicated cold competitor concentration divided by the total probe (i.e. free DNA and DNA–DprA complexes). The standard deviations from 3 independent experiments are indicated by bars.

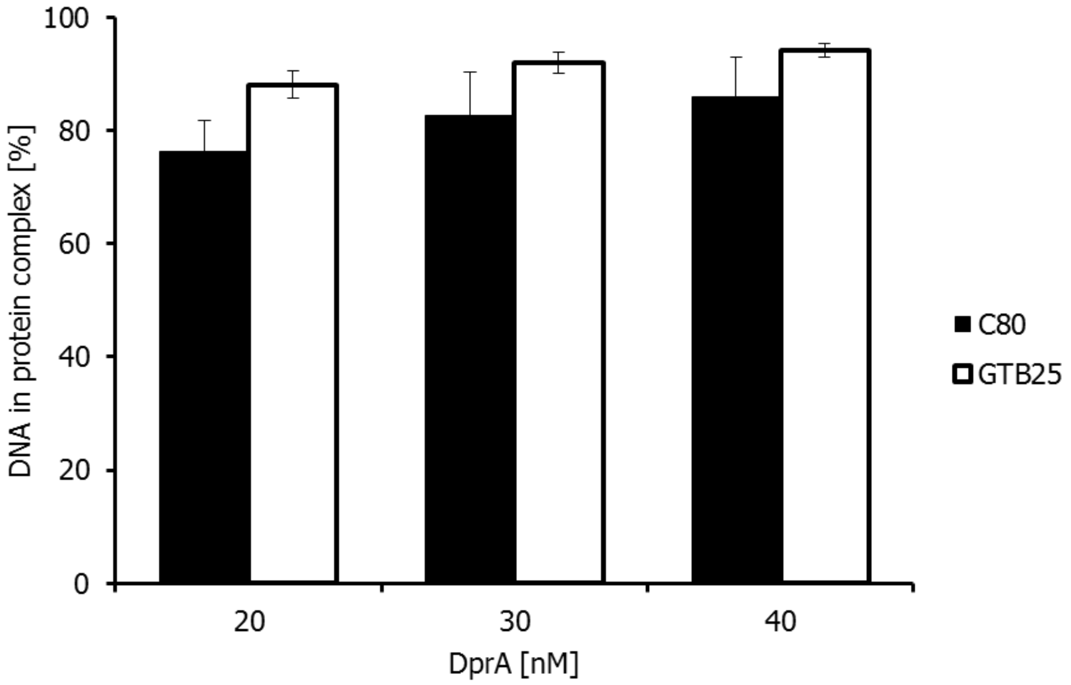

Figure S7. Quantitation of the DNA binding activity DprA of the figure 5A Lanes 2-7: where increasing concentration of recombinant DprA protein (nM) incubated with 1000 CPM/μl of the [γ^{32}P]ATP labeled C80nt (i) and GTB25(ii). DprA binding activity is expressed as a fraction of DNA–DprA complexes at the indicated cold competitor concentration divided by the total probe (i.e. free DNA and DNA–DprA complexes). The standard deviations from 3 independent experiments are indicated by bars.

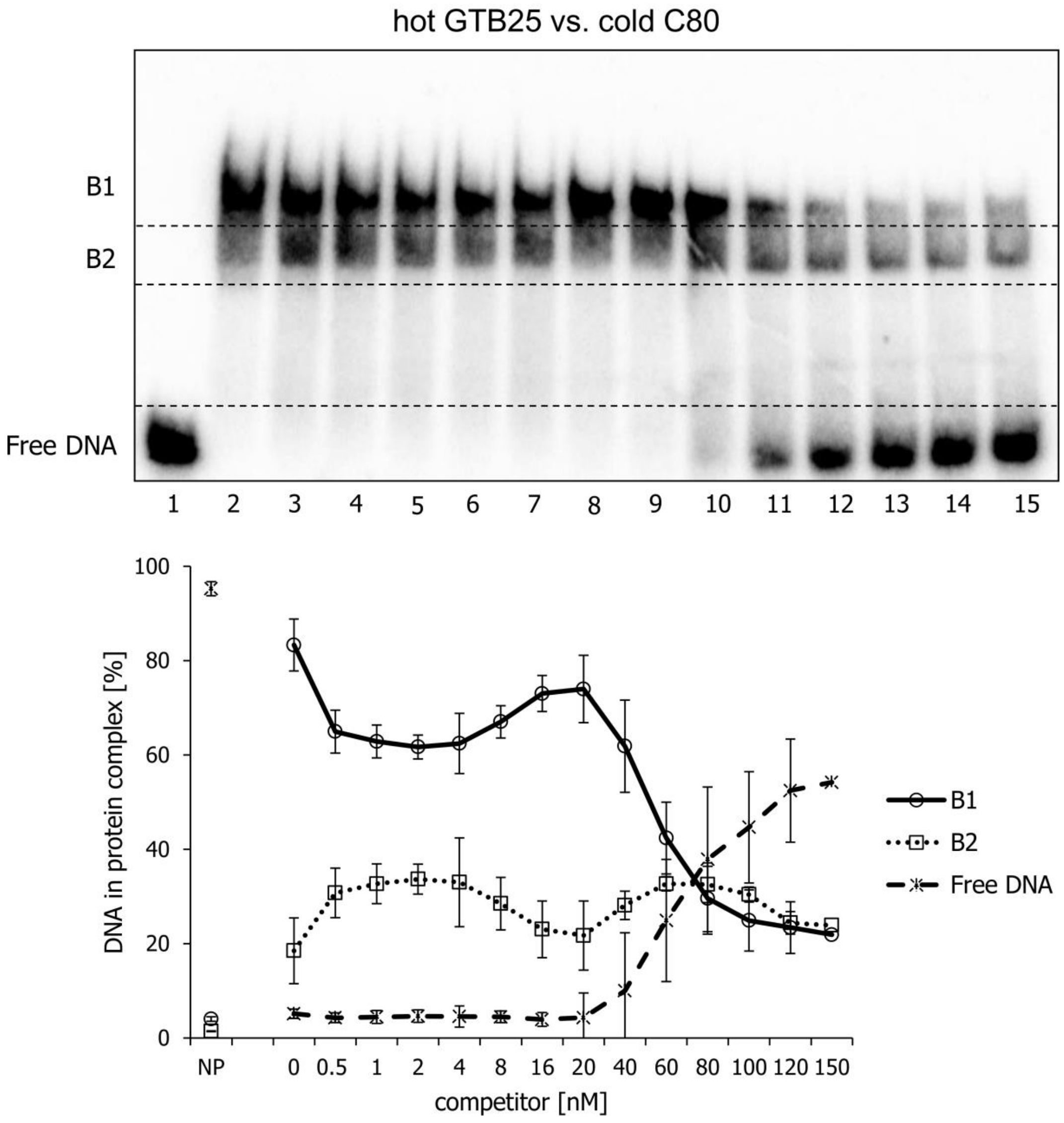

Figure S8. Representative gel images (upper panels) **and quantitation** (lower panels) **of the competitive EMSA:** where 30 nM recombinant DprA protein incubated with 1000 CPM/µl of $[\gamma^{32}P]$ATP labeled (hot) GTB25 and competed out with increasing concentration of cold C80. DprA binding activity is expressed as a fraction of DNA–DprA complexes at the indicated cold competitor concentration divided by the total probe (i.e. free DNA and DNA–DprA complexes). The standard deviations from 3 independent experiments are indicated by bars. NP – no protein, NC – no competitor DNA, B1- band one, and B2 - band 2.

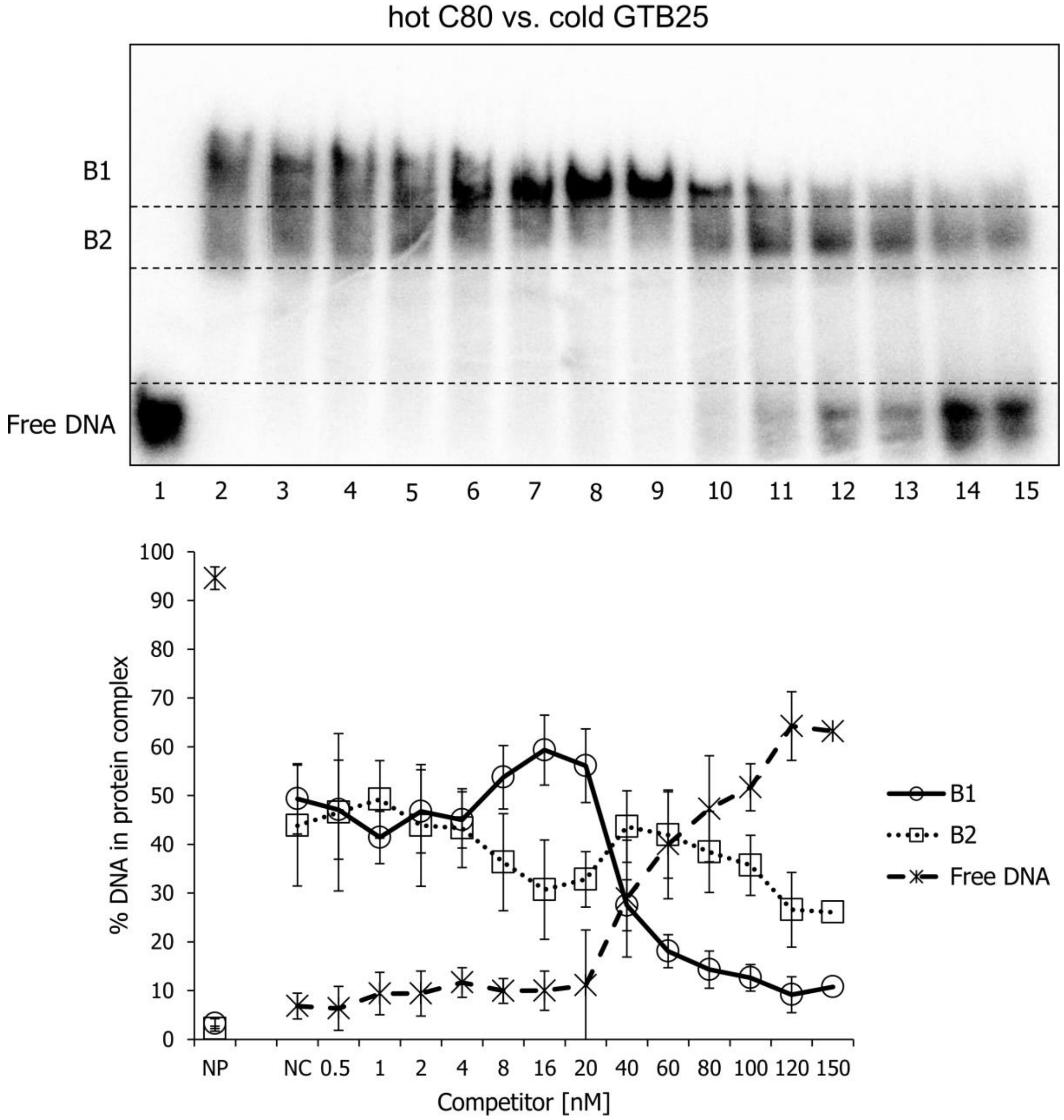

Figure S9. Representative gel images (upper panels) **and quantitation** (lower panels) **of the competitive EMSA of DprA:** where 30 nM recombinant DprA protein incubated with 1000 CPM/µl of [γ^{32}P]ATP labeled C80 and competed out with increasing concentration of cold GTB25. DprA binding activity is expressed as a fraction of DNA–DprA complexes at the indicated cold competitor concentration divided by the total probe (i.e. free DNA and DNA–DprA complexes). The standard deviations from 3 independent experiments are indicated by bars. NP – no protein, NC – no competitor DNA, B1- band one, and B2 - band 2.

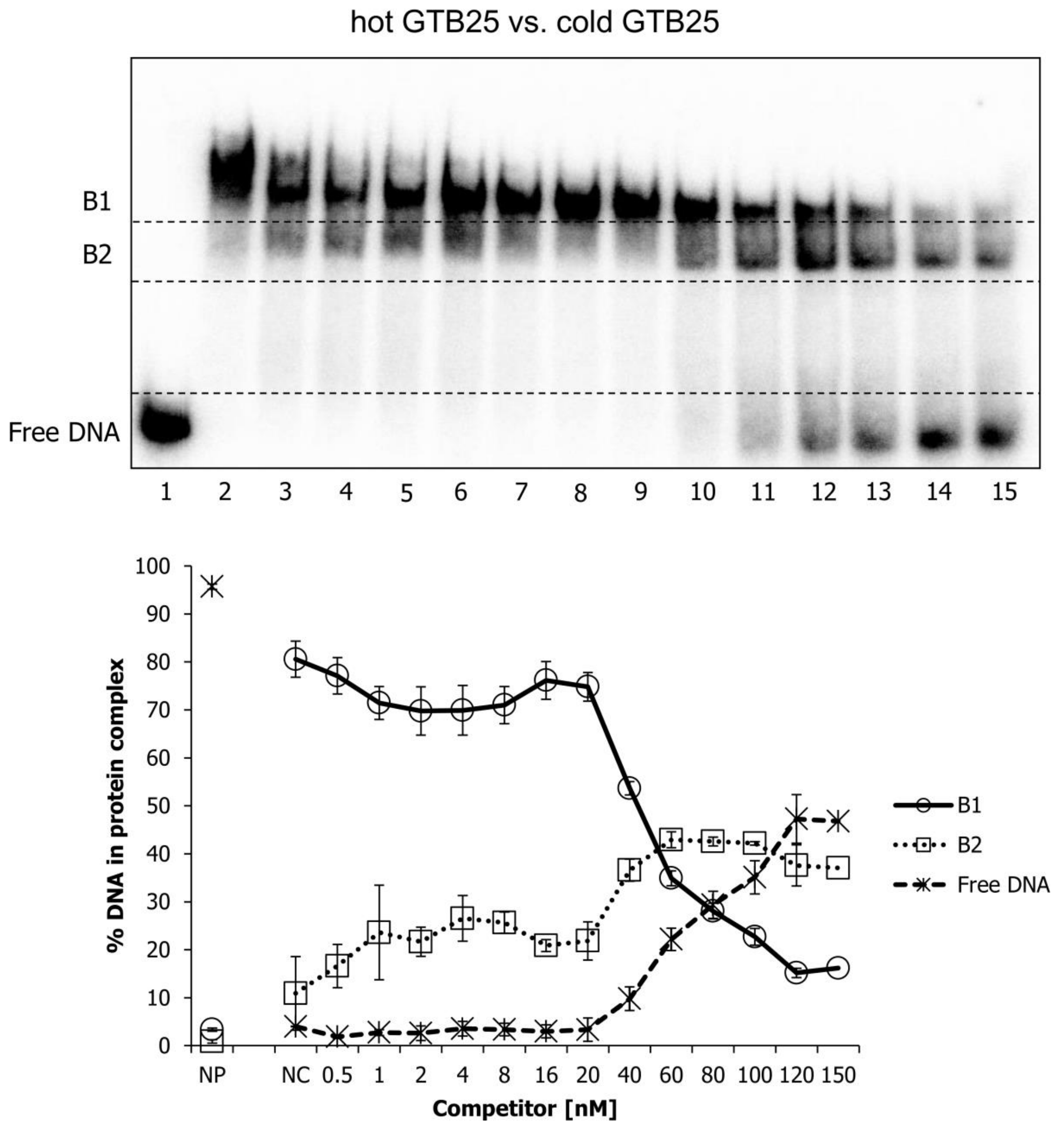

Figure S10. Representative gel images (upper panels) **and quantitation** (lower panels) **of the competitive EMSA:** where 30 nM recombinant DprA protein incubated with 1000 CPM/µl of [γ^{32}P]ATP labeled (GTB25) and competed out with increasing concentration of cold GTB25. DprA binding activity is expressed as a fraction of DNA–DprA complexes at the indicated cold competitor concentration divided by the total probe (i.e. free DNA and DNA–DprA complexes). The standard deviations from 3 independent experiments are indicated by bars. NP – no protein, NC – no competitor DNA, B1- band one, and B2 - band 2.

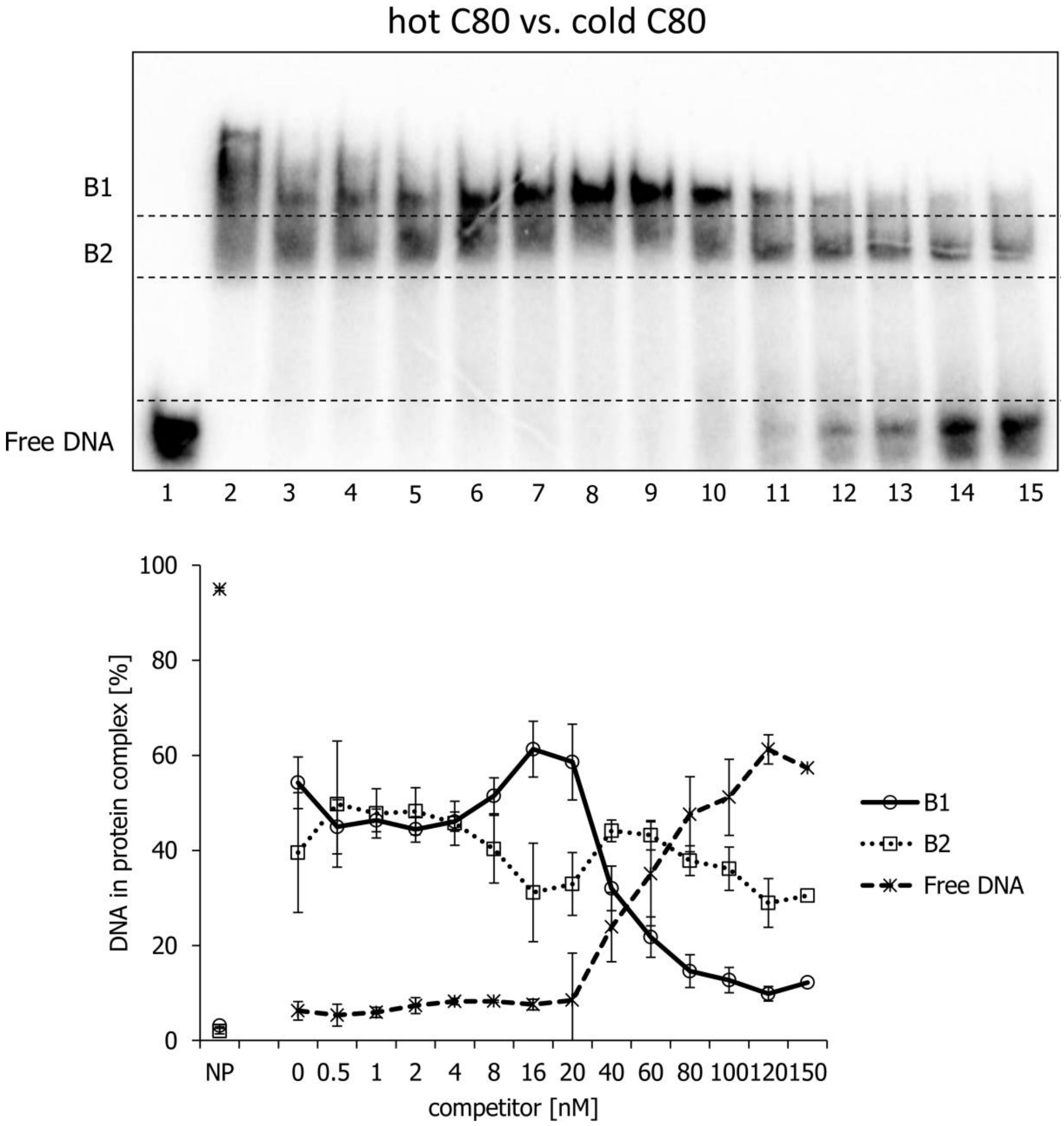

Figure S11. Representative gel images (upper panels) **and quantitation** (lower panels) **of the competitive EMSA:** where 30 nM recombinant DprA protein incubated with 1000 CPM/µl of [γ^{32}P]ATP labeled C80 and competed out with increasing concentration of cold C80. DprA binding activity is expressed as a fraction of DNA–DprA complexes at the indicated cold competitor concentration divided by the total probe (i.e. free DNA and DNA–DprA complexes). The standard deviations from 3 independent experiments are indicated by bars. NP – no protein, NC – no competitor DNA, B1- band one, and B2 - band 2.
